# Jiawei Xianglian Decoction (JWXLD), a Traditional Chinese Medicine (TCM), Alleviates CPT-11-Induced Diarrhea in Mice

**DOI:** 10.1155/2020/7901231

**Published:** 2020-03-12

**Authors:** Jinhua Lu, Zechen Lin, Siyu Huang, Yiwei Shen, Jing Jiang, Shengyou Lin

**Affiliations:** ^1^Department of Traditional Chinese Comprehensive Medical Oncology, Hangzhou Cancer Hospital, Hangzhou, China; ^2^Oncology Department, Dingqiao Branch of GuangXing Hospital Affiliated to Zhejiang Chinese Medical University, Hangzhou, China; ^3^Zhejiang Chinese Medical University, Hangzhou, China; ^4^Oncology Department, GuangXing Hospital Affiliated to Zhejiang Chinese Medical University, Hangzhou, China

## Abstract

Irinotecan (CPT-11) is used for therapy of various cancers. However, it has several serious adverse reactions such as diarrhea which is caused by SN-38, the active form of CPT-11. This study aimed to evaluate the effectiveness of Jiawei xianglian decoction (JWXLD), which has been widely used for the treatment of diarrhea in China. In this study, a mouse model with delayed diarrhea was generated by CPT-11. Real-time PCR and enzyme-linked immunosorbent assay (ELISA) were performed to explore intestinal microflora and inflammatory cytokine. Hematoxylin and eosin (H&E) staining was used to analyze tissue morphology. We found that 0.12, 0.23, and 0.46 g JWXLD significantly reduced the severity of CPT-11-induced diarrhea. The levels of *Lactobacillus* (*Lacto*) and *Bifidobacterium* (*Bifid*) were significantly downregulated by CPT-11, and these effects can be reversed by JWXLD treatment. Furthermore, JWXLD was observed to decrease the activity of *β*-glucuronidase (*β*-GD). Histopathological data showed that CPT-11 induced severe intestinal mucosal injury, which was characterized as grade 6, and JWXLD significantly alleviated the injury. In addition, CPT-11 increased the productions of tumor necrosis factor-alpha (TNF-*α*), tumor necrosis factor-beta (TNF-*β*), interleukin-6 (IL-6), and interleukin-1 (IL-1), but decreased interleukin-15 (IL-15), interleukin-7 (IL-7), and uridine diphosphate-glucuronosyltransferase 1A1 (UGT1A1). In conclusion, JWXLD can counteract these effects caused by CPT-11 treatment. JWXLD could alleviate CPT-11-induced diarrhea.

## 1. Introduction

Irinotecan (CPT-11), an agent of camptothecin, was firstly isolated from *Camptotheca acuminata* (family: Nyssaceae) in the early 1960s [1]. CPT-11 exerts a broad spectrum of antitumor activity and has been used for the treatment of many kinds of cancer, including colon cancer [2–4]. However, it can cause serious adverse reactions, such as transient neutropenia and delayed diarrhea. As reported, transient neutropenia occurs in approximately 80% of patients, and delayed diarrhea occurs in approximately 87% of patients [5]. Blocking DNA replication and transcription via inhibiting topoisomerase-1 has been demonstrated to be the pharmacological effect of CPT-11 [6]. The metabolic pathway of CPT-11 is complex. At first, CPT-11 is converted into its active metabolite SN-38 by carboxylesterase (CES) 1 and CES2. SN-38 is subsequently metabolized into SN-38 glucuronide (SN-38G) by uridine diphosphate-glucuronosyltransferase 1A1 (UGT1A1) [7]. The SN-38G can be excreted into the bile and deconjugated to SN-38 by *β*-glucuronidase (*β*-GD) in the intestine, leading to the accumulation of SN-38 [8, 9]. This SN-38 is considered to induce severe diarrhea [10]. Delayed diarrhea is considered to be one of the dose-limiting factors of CPT-11, so it is urgent to find therapies to control delayed diarrhea induced by CPT-11.

To date, various experimental agents and strategies are employed to treat CPT-11-induced delayed diarrhea, including inhibition of SN-38 production, promotion of SN-38 adsorption, and suppression of *β*-GD activity. For example, neomycin [11], cholestyramine/levofloxacin [12], and cefpodoxime [13] are used to alleviate CPT-11-induced delayed diarrhea by inhibiting the production of SN-38. Activated charcoal has been reported to prevent irinotecan-induced diarrhea [14]. Although these strategies can alleviate diarrhea, they cannot completely block delayed diarrhea due to the complex pathogenesis. Traditional Chinese medicines (TCMs) have been used to reduce the side effects of cancer-related medicines for thousands of years [15]. Jiawei xianglian decoction (JWXLD) is composed of stir-fried *Coptis chinensis* Franch (Ranunculaceae) (chao huanglian in Chinese), *Aucklandia lappa* (Compositae) (muxiang in Chinese), *Sargentodoxa cuneate* Rehder and E.H.Wilson (Lardizabalaceae) (daxueteng in Chinese), *Taraxacum mongolicum* Hand.-Mazz (Compositae) (pugongying in Chinese), *Portulaca oleracea* L. (Portulacaceae) (machixian in Chinese), and *Euphorbia humifusa* Willd (Euphorbioideae) (dijincao in Chinese). Gegen Qinlian decoction, one of the active component is *Coptis chinensis*, has been shown to alleviate inflammation through suppressing TLR4/NF-*κ*B signaling in acute ulcerative colitis [16]. *Portulaca oleracea* [17]*, Euphorbia humifusa* [18], and *Aucklandia lappa* [19] have been reported to play a role in treating with inflammation. JWXLD is a clinically used drug in China, and it has curative effects in the treatment of diarrhea. However, the exact role of JWXLD in chemotherapy-associated diarrhea remains unclear.

In the current study, we used CPT-11, a frequently used chemotherapeutic drug, to generate diarrhea model and explore how JWXLD can alleviate diarrhea. Our results showed that JWXLD can alleviate diarrhea induced by CPT-11.

## 2. Methods

### 2.1. Materials and Reagents

CPT-11 was purchased from Meilun Biotechnology (Dalian, China). Loperamide was purchased from Xian Janssen Pharmaceutical (Xian, China).

### 2.2. Preparation of JWXLD

Stir-fried *Coptis chinensis*, *Aucklandia lappa*, *Sargentodoxa cuneate*, *Taraxacum mongolicum*, *Portulaca oleracea*, and *Euphorbia humifusa* were decocted at a rate of 2 : 3 : 5 : 4 : 10 : 5 in dry weight. After filtration, the complex was concentrated to 100%, which means that 1 g raw drugs per milliliter. The detection was kept at 4°C in a refrigerator for further use.

### 2.3. Animals and Treatments

48 BALB/C mice (weighing 20 ± 2 g) were obtained from the Shanghai Laboratory Animal Research Center (Shanghai, China). The mice were housed under 12 h light/dark cycle at 20°C–23°C and 40–60% humidity with free access to food and water. After 1 week of normal diet, the mice were randomly divided into 6 groups (control, CPT-11 + loperamide, CPT-11, CPT-11 + 0.12 g JWXLD, CPT-11 + 0.23 g JWXLD, and CPT-11 + 0.46 g JWXLD) with 8 rats in each group. JWXLD was given to the mice through gavage once a day for 7 days, starting from day 0 to day 7. All the groups except the control group were given 75 mg/kg CPT-11 through intraperitoneal injection once a day for 4 days, starting from day 1 to day 5. The loperamide group was given 0.23 g loperamide through gavage once a day for 7 days, starting from day 0 to day 7. The control group was given the same amount of normal saline instead. All experiments were performed in accordance with the National Institutes of Health Guidelines for Animal Research and approved by the Ethics Committee of the Institute of Zhejiang Chinese Medical University.

On the 8th day, mice feces were collected, and all the mice were sacrificed. Ileum and liver of each mouse were cut and kept in a 10% formalin solution or at −80°C in the refrigerator for further experiments.

### 2.4. Assessment of Diarrhea

On the third day following the final administration of CPT-11, mice feces were collected to analyze the degree of diarrhea. The severity of diarrhea was scored as follows [20]: 0, normal feces; 1, soft feces or small black feces; 2, wet and unformed feces; and 3, watery feces with severe perianal staining of the coat.

### 2.5. Real-Time PCR

For the analysis of intestinal microflora, 0.2 g mice normal feces and 1.5 ml phosphate-buffered saline (PBS) were added, mixed for 5 min, and then centrifuged at 1000 rpm for 10 min. The supernatants were treated 3 times as described above. For the last time, the supernatants were centrifuged at 14000 rpm for 10 min, and the sediments were retained. Next, the sediments were washed 4 times with 1 ml PBS. The bacteria were broken with Triton X-100, washed with phenol/chloroform, and deposited with cold ethanol. After being dried at room temperature, bacterial DNA was dissolved with sterile water.

The amplification of intestinal microflora DNA including *Lactobacillus* (*Lacto*), *Bifidobacterium* (*Bifid*), and *Escherichia coli* (*E. coli*) was performed on a TaKaRa PCR amplifier (Dalian, China) using SYBR Green mix assay (TaKaRa, Dalian, China). The procedure is as follows: 95°C for 10 min; 95°C for 15 sec, and 60°C for 45 sec with 40 cycles, at last 95°C for 15 sec and 60°C for 1 min. The DNA copy number was measured by the standard curve.

*Lacto*
     Primer F: 5′-ACGGGAGGCAGCAGTAGGGA-3′
     Primer R: 5′-AGCCGTGACTTTCTGGTTGATT-3′
*Bifid*
     Primer F: 5′-GATTCTGGCTCAGGATGAACGC-3′
     Primer R: 5′-CTGATAGGACGCGACCCCAT-3′
E. *coli*
     Primer F: 5′-CATGCCGCGTGTATGAAGAA-3′
     Primer R: 5′-CGGGTAACGTCAATGAGCAAA-3′

For the relative expression level of target genes, the total RNA of liver tissues was extracted using Trizol reagent kit (Invitrogen, Carlsbad, CA, USA) and reverse transcribed to cDNA using cDNA synthesis kit (Promega, Madison, WI, USA). The amplification system was used as described above. Relative quantification of the gene expression level was presented using the comparative Ct method (2^−ΔCt^) and GAPDH as the internal reference gene.

UGT1A1
       Primer F: 5′-AGTCTGGCACTCTGCTTTC-3′
       Primer R: 5′-GTGTCACAGCCTCATCTCTTC-3′
GAPDH
       Primer F: 5′-CTGCCCAGAACATCATCC-3′
       Primer R: 5′-CTCAGATGCCTGCTTCAC-3′

### 2.6. Analysis of *β*-GD

To draw the p-nitrophenol (PNP) standard curve, PNP (Sigma, St. Louis, MO, USA) was diluted into 320, 160, 80, 40, 20, and 0 nmol/ml. Then, 1 ml PBS was added to each tube, and the absorbance at 405 nm was measured by an auto-microplate reader (Bio-Rad Laboratories, Inc., Hercules, CA, USA). The optical density (OD) value and PNP concentration value were used to draw the standard curve.

For the analysis of *β*-GD, 0.1 g mice feces and 2 ml PBS were added, mixed for 5 min, and then added 0.1 ml nitrophenyl *β*-D-glucuronide (Sigma, St. Louis, MO, USA). After incubation for 20 min at 30°C, the mix was centrifuged at 3000 rpm for 10 min. The supernatants were measured at 405 nm using an auto-microplate reader. The activity of *β*-GD was calculated by the following equation.

(1) $$\begin{array}{c} \beta -\text{GD}\left(\text{nmol/g}\times \text{min}\right)=\frac{K\left(\text{PNP concentration}\right)\times 2\text{ml}}{0.1\text{g}\left(\text{the weight of feces}\right)\times 20\min}, \end{array}$$

where *K* is the concentration of PNP.

### 2.7. Hematoxylin and Eosin (H&E) Staining

After fixing in a 10% formalin solution for 48 h, the ileum tissue was embedded with paraffin and cut into sections (5 mm; Leica RM2125, Germany). Sections were stained with H&E according to standard methods. Then, a light microscope (Olympus, Tokyo, Japan) was used to collect the images at ×200 magnification. The degree of intestinal mucosal injury was graded according to Chiu et al. [21] as follows: 1, normal mucosal villi; 2, development of a subepithelial space, usually at the apex of the villi with capillary congestion; 3, extension of the subepithelial space with moderate lifting of the epithelial layer from the lamina propria; 4, massive epithelial lifting down the sides of the villi and ulceration at the villous tips; 5, denuded villi with dilated capillaries and increased cellularity of the lamina propria; and 6, degradation and disintegration of the lamina propria, hemorrhage, and ulceration.

### 2.8. Enzyme-Linked Immunosorbent Assay (ELISA)

First, frozen ileum samples were homogenized in iced PBS. ELISA kits (Meso Scale Discovery, Rockville, MD, USA) were used to determine IL-1, IL-6, IL-7, IL-15, TNF-*α*, and TNF-*β* from ileum samples according to the manufacturer's instructions.

### 2.9. Western Blot

First, frozen liver samples were homogenized in RIPA lysis buffer (Solarbio, Beijing, China) and centrifuged at 12000 rpm for 10 min at 4°C. Then, the supernatant was separated by 10–15% SDS-PAGE gel (the concentration of the gel was decided according to the protein molecular weight), followed by transferring to nitrocellulose membranes (Millipore Corp., Bedford, MA, USA). The membranes were blocked with TBST containing 5% milk for 1 h at room temperature and incubated with UGT1A1 (Abcam, 1 : 700 dilution) and GAPDH (Abcam, 1 : 2000 dilution) primary antibody at 4°C overnight. Then, the membranes were washed 3 times with TBST and incubated with secondary antibodies for 1 h. After 2 times washing, the brands were developed by an enhanced chemiluminescence (ECL) kit (Millipore, Burlington, MA, USA) and collected with the scanner. GAPDH was used as an internal control.

### 2.10. Statistical Analysis

All values are showed as mean ± SD. One-way analysis of variance (ANOVA) was used to analyze data of different groups. *P* < 0.05 was considered significant.

## 3. Results

### 3.1. Effect of JWXLD on the Diarrhea Score in the CPT-11-Induced Delayed Diarrhea Model

To generate a CPT-11-induced delayed diarrhea mouse model, 75 mg/kg CPT-11 was injected once a day for 4 days [22]. On the third day following the final administration of CPT-11, mice feces were collected to analyze the diarrhea degree. We found that CPT-11 caused severe diarrhea, and all the three doses of JWXLD significantly reduced the severity of diarrhea induced by CPT-11, and 0.23 g of JWXLD exhibited the best effect. Loperamide, a frequently used antidiarrheic drug, also prominently alleviated diarrhea (Table 1).

### 3.2. JWXLD Increased Intestinal Microflora Contents in the CPT-11-Induced Delayed Diarrhea Model

**Figure 1:**
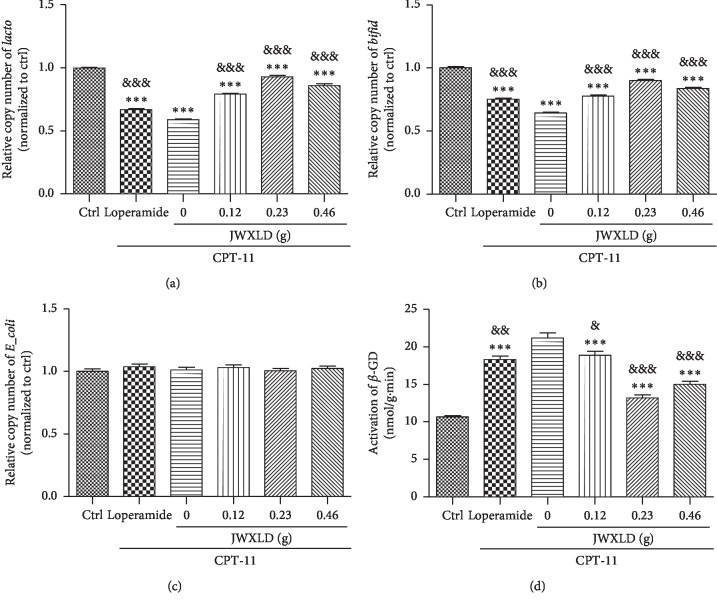
Effect of JWXLD on intestinal microflora in the CPT-11-induced delayed diarrhea model. A, B, C: DNA copy numbers of *Lacto*, *Bifid* and *E. coli* were measured by real-time PCR. D: Activity of *β*-GD was measured after treated with CPT-11 and/or different doses of JWXLD. ^*∗∗∗*^, *P* < 0.001 vs control; &&&, *P* < 0.001 vs. CPT-11. (a) *Lacto*. (b) *Bifid*. (c) *E. coli* (d). *β*-GD.

As reported, intestinal microflora plays a role in the intestinal toxicity of irinotecan. *E. coli* is reported to produce *β*-GD [23], *Lacto* is suggested to inhibit *β*-GD [24], and *Bifid* is reported to have protective properties towards the gut mucosal barrier [25]. In order to understand the role of JWXLD, intestinal microflora including *Lacto*, *Bifid*, *E. coli* and *β*-GD were measured. As shown in Figure 1, levels of *Lacto* and *Bifid* were significantly decreased after treated with CPT-11 and all the three doses of JWXLD markedly increased these contents compared to CPT-11, and 0.23 g of JWXLD exhibited the best effect. While the level of *E. coli* was not changed at all. We also found that the activity of *β*-GD was inhibited by JWXLD compared with CPT-11, and 0.23 g of JWXLD showed the highest inhibition (Figure 1). Loperamide showed the similar effects as JWXLD.

### 3.3. JWXLD Relieved Pathogenesis in the CPT-11-Induced Delayed Diarrhea Model

**Figure 2:**
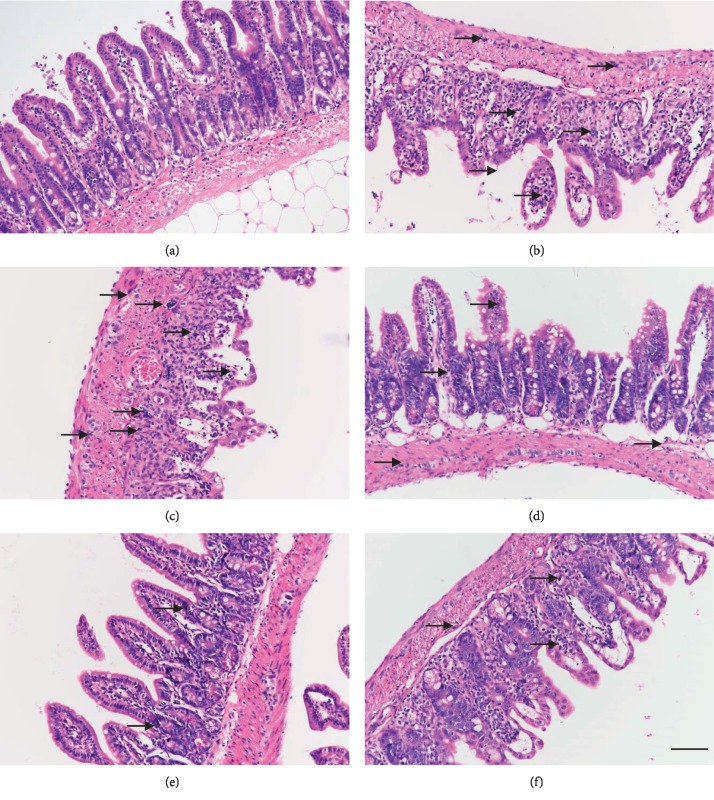
H&E staining of ileum tissues. (a) Control group; (b) CPT-11 + loperamide group; (c) CPT-11 group; (d) CPT-11 + 0.12 g JWXLD group; (e) CPT-11 + 0.23 g JWXLD group; (f) CPT-11 + 0.46 g JWXLD group. The black arrows indicate infiltration by inflammatory cells. Scale: 100 *μ*m.

The condition of the ileum tissue was analyzed by HE staining. CPT-11 induced the shortening of the villi, the damage of crypt cells, and infiltration of inflammatory cells into the lamina propria (Figure 2(c)) and characterized intestinal mucosal injury of grade 5.87 (Table 2). JWXLD significantly alleviated the histopathological alterations which were observed in the CPT-11 group (Figure 2(d)–2(f)) (grade 4, 3, 4). Treatment with 0.23 g of JWXLD increased the villi, reduced the infiltration of inflammatory cells and repaired crypt cells and architecture (Figure 2(e)) (grade 3). In addition, loperamide showed the similar effects as JWXLD (grade 4).

### 3.4. JWXLD Inhibited the Production of Proinflammatory Cytokines but Promoted Anti-Inflammatory Cytokines in CPT-11-Induced Delayed Diarrhea Model

**Figure 3:**
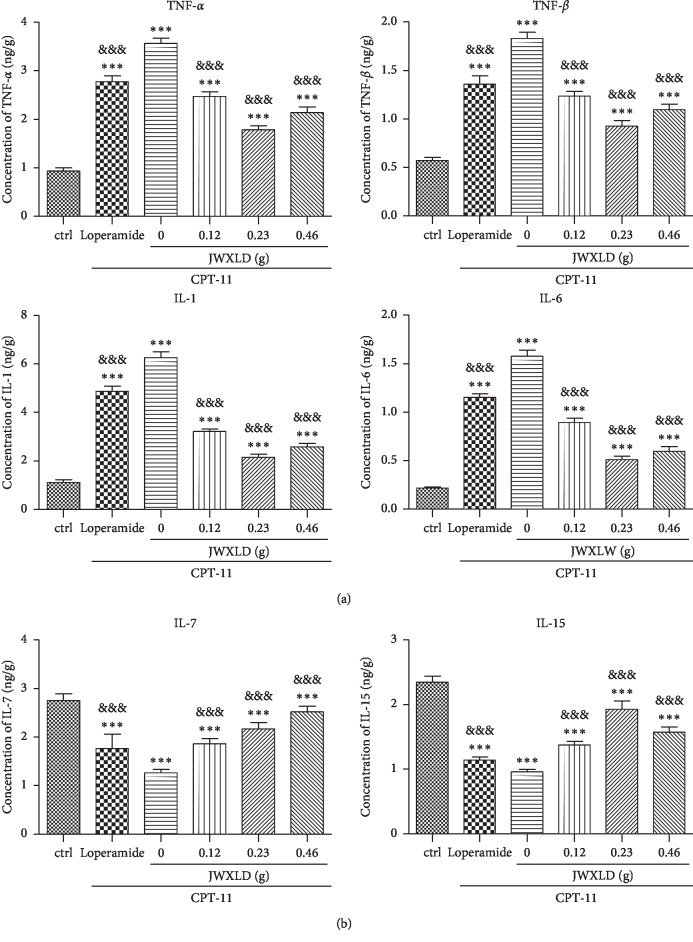
(a, b) JWXLD inhibited the production of proinflammatory cytokines in the CPT-11-induced delayed diarrhea model. The production levels of TNF-*α*, TNF-*β*, IL-6, IL-1, IL-15 and IL-7 were measured using ELISA assay. ^*∗∗∗*^, *P* < 0.001 vs. control; &&&, *P* < 0.001 vs. CPT-11.

Pro- and anti-inflammatory cytokine expressions have been showed to play a key role in the pathogenesis of diarrhea [22, 26, 27]. As shown in Figure 3(a), CPT-11 induced high production levels of TNF-*α*, TNF-*β*, IL-6, and IL-1 of ileum tissues. All the three doses of JWXLD markedly inhibited the production of proinflammatory cytokines induced by CPT-11, and 0.23 g of JWXLD exhibited the best effect. On the contrary, CPT-11 significantly decreased the ileum tissue level of IL-15 and IL-7, and these inhibitions could be counteracted by JWXLD (Figure 3(b)). Loperamide showed the similar effects as JWXLD.

### 3.5. JWXLD Overcame the Inhibition of UGT1A1 Induced by CPT-11

**Figure 4:**
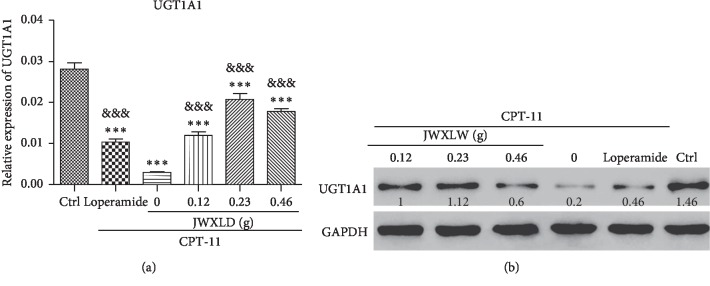
JWXLD reverses the inhibition of UGT1A1 induced by CPT-11. The expression level of liver UGT1A1 was measured by real-time PCR (upper panel) and Western blot (lower panel) (*n* = 8). ^*∗∗∗*^, *P* < 0.001 vs. control; &&&, *P* < 0.001 vs. CPT-11.

As reported [28], UGT1A1 mediates conversion of the active SN-38 to inactive SN-38G and subsequently alleviates delayed diarrhea induced by SN-38. Then, we analyzed the expression level of UGT1A1 after treatment with CPT-11 and/or JWXLD. Interestingly, we observed that CPT-11 inhibited the expression of UGT1A1 at mRNA and protein levels, and this inhibition can be partly reversed by JWXLD and loperamide (Figure 4).

## 4. Discussion

As a frequent side effect in patients receiving chemotherapy, chemotherapy-induced diarrhea (CID) was reported as high as 50–80% in patients treated with irinotecan [29]. Germ-free mice showed more resistance to lethal dosage of CPT-11 by 2.5 folds than that of holoxenic mice [30]. The underlying mechanism contributing to CPT-11-induced diarrhea was shown that SN-38 G can be hydrolyzed to SN-38 by bacterial *β*-GD, thus leading to intestinal toxicity. Thus, inhibiting the activity of *β*-GD is expected to alleviate diarrhea induced by CPT-11 [29].

Recently, the inhibition of AIM2 by thalidomide, an inflammasome inhibitor, is found to alleviate CPT-11-induced intestinal toxicity without compromising its anticancer efficacy [31]. In addition, one method to protect the intestine from damage induced by reactivation of SN-38 is to inhibit bacterial activity of the intestinal lumen. More severe intestinal histology injury and higher levels of proinflammatory IL-1*β* and TNF-*α* cytokines are observed in conventional mice after irinotecan treatment compared with germ-free mice [32]. D-Saccharic acid 1.4-lactone (SAL) was reported by Fittkau et al. which has the effect to reduce irinotecan-induced mucosal damage in rats [33]. Similarly, another group found inhibitor 1, a specific potent bacterial *β*-GD inhibitor, significantly suppressed CPT-11-induced diarrhea and intestinal damage in mice [34]. Unexpectedly, pharmacokinetics of SN-38 in plasma which used to determine antitumor efficacy of CPT-11, may be simultaneously influenced by such inhibition. Gupta et al. indicated that SN-38 was absorbed from the intestine as the dominant peak of SN-38 in serum was observed after intravenous infusion of CPT-11 in patients [35]. These findings indicate the enterohepatic recirculation of SN-38. Based on those studies, inhibition of bacterial *β*-GD activity could relieve the antitumor efficacy of CPT-11 by increasing the level of SN-38G and reducing the reabsorption level of SN-38 and in the intestine.

In this study, we explored that JWXLD, a traditional Chinese herbal decoction, could alleviate diarrhea induced by CPT-11. Firstly, 75 mg/kg CPT-11 was treated once a day for 4 days, and severe diarrhea was developed by the third day following the final administration. These results indicated that CPT-11 can cause delayed diarrhea (grade 2.56) [36, 37], and different doses of JWXLD can alleviate diarrhea (Table 1). However, 0.23 g of JWXLD rather than 0.46 g of JWXLD exhibited the best effect (grade 0.12), which might be caused by the following reason. A traditional Chinese herbal decoction usually has a dose-dependent curve in a range of doses, and 0.46 g of JWXLD may be above this range so that it does not match the dose-dependent curve. Next, we found that CPT-11 decreased levels of *Lacto* and *Bifid*, but failed to regulate the level of *E. coli* (Figure 1). *Lacto* has been found to be inhibited after treatment with CPT-11 [38] and *Bifid longum* (*B. longum*) has been found to prevent small intestinal mucositis induced by CPT-11 [39]. In this study, JWXLD was observed to increase the levels of *Lacto* and *Bifid* compared with CPT-11. In agreement, we found that JWXLD treatment significantly inhibited *β*-GD upon treatment with higher dosage (Figure 1). Hepatic UGT1A1 can glucuronidate SN-38 to inactivated SN-38G [15]. In this study, we observed increase of UGT1A1 with treatment of JWXLD in Figure 4. Collectively, JWXLD treatment inhibited bacterial *β*-GD and increased UGT1A1 and finally prevented diarrhea induced by CPT-11 (Figure 2). In consistent, bacterial contents of *Lacto* and *Bifid* were increased as well (Figure 1). Furthermore, proinflammatory (TNF-*α*, TNF-*β*, IL-1, and IL-6) were suppressed supporting its protective effects of JWXLD in CPT-11-induced diarrhea (Figure 3). CPT-11 has been reported to significantly increase the level of inflammatory cytokines such as TNF-*α*, IL-1, and IL-6 in the rat colon [40], which is consistent with our findings. However, how JWXLD alleviates inflammation is still not clear. Fang et al. reported CPT-11-induced elevation of bile acids which potentiates suppression of IL-10 expression [41]. Kon et al. revealed CPT-11-induced delayed diarrhea develops via reduced aquaporin-3 expression in the colon [40]. Li et al. found CPT-11 activates NLRP3 inflammasome through JNK and NF-*κ*B signaling pathway [42, 43]. For clinical application purpose, what are the effects of JWXLD on antitumor efficacy of CPT-11 need be explored more in future.

## 5. Conclusion

In summary, we showed JWXLD, an important component of TCMs, prevents CPT-11-induced diarrhea.

## Figures and Tables

**Table 1:** Diarrhea score was analyzed after treatment with different doses of JWXLD in CPT-11-induced delayed diarrhea mouse model.

		CPT-11 (75 mg/kg)				
			JWXLD (g)			

Group	Control	Loperamide	0	0.12	0.23	0.46

Average values of diarrhea scores	0	1.87^*∗#*^	2.56^*∗*^	1.12^*∗#*^	0.12^*∗#*^	0.82^*∗#*^

Data represent average values of diarrhea scores (*n* = 8). ^*∗*^, *P* < 0.05 compared to control mice; ^#^, *P* < 0.05 compared to the mice treated with CPT-11.

**Table 2:** Intestinal mucosal injury grade was analyzed after administration of CPT-11 of mice treated with different doses of JWXLD.

		CPT-11 (75 mg/kg)				
			JWXLD (g)			
Group	Control	Loperamide	0	0.12	0.23	0.46

Average intestinal mucosal injury grade	1.25	4.75^*∗#*^	5.87∗	4.25^*∗#*^	2.75^*∗#*^	3.25^*∗#*^

Data represent average values of intestinal mucosal injury grade (*n* = 8). ^*∗*^*P* < 0.05 compared to control mice; ^#^*P* < 0.05 compared to the mice treated with CPT-11.

## Data Availability

The data used to support the findings of this study are available from the corresponding author upon request.
